# Metabolic Control and Illness Perceptions in Adolescents with Type 1 Diabetes

**DOI:** 10.1155/2016/3486094

**Published:** 2015-11-22

**Authors:** Line Wisting, Lasse Bang, Henrik Natvig, Torild Skrivarhaug, Knut Dahl-Jørgensen, Bryan Lask, Øyvind Rø

**Affiliations:** ^1^Regional Department for Eating Disorders, Division of Mental Health and Addiction, Oslo University Hospital, P.O. Box 4956, Nydalen, 0424 Oslo, Norway; ^2^Department of Psychology, University of Oslo, Forskningsveien 3A, 0373 Oslo, Norway; ^3^The Norwegian Childhood Diabetes Registry, Department of Pediatric Medicine, Oslo University Hospital, P.O. Box 4956, Nydalen, 0424 Oslo, Norway; ^4^Department of Pediatric Medicine, Oslo University Hospital, P.O. Box 4956, Nydalen, 0424 Oslo, Norway; ^5^Faculty of Medicine, University of Oslo, P.O. Box 1078, Blindern, 0316 Oslo, Norway; ^6^Oslo Diabetes Research Centre, P.O. Box 4956, Nydalen, 0424 Oslo, Norway; ^7^Rhodes Farm Clinic, The Ridgeway, Barnet, London NW7 1RH, UK; ^8^Gt. Ormond St. Hospital for Children, Great Ormond Street, London WC1N 3JH, UK; ^9^Department of Psychology, University of Exeter, Perry Road, Exeter EX4 4QG, UK; ^10^Division of Mental Health and Addiction, Institute of Clinical Medicine, University of Oslo, P.O. Box 1039, Blindern, 0315 Oslo, Norway

## Abstract

*Background*. Disturbed eating behavior and psychosocial variables have been found to influence metabolic control, but little is known about how these variables interact or how they influence metabolic control, separately and combined. *Objective*. To explore associations between metabolic control (measured by HbA1c) and eating disorder psychopathology, coping strategies, illness perceptions, and insulin beliefs in adolescents with type 1 diabetes. *Methods*. A total of 105 patients (41.9% males) with type 1 diabetes (12–20 years) were interviewed with the Child Eating Disorder Examination. In addition, self-report psychosocial questionnaires were completed. Clinical data, including HbA1c, was obtained from the Norwegian Childhood Diabetes Registry. *Results*. Significant gender differences were demonstrated. Among females, HbA1c correlated significantly with eating restriction (.29, *p* < .05), the illness perception dimensions *consequences, personal control, coherence,* and *concern* (ranging from .33 to .48), and the coping strategy *ventilating negative feelings* (−.26, *p* < .05). Illness perception *personal control* contributed significantly to HbA1c in a regression model, explaining 23% of the variance among females (*β* .48, *p* < .001). None of the variables were significantly associated with HbA1c among males. *Conclusions*. Illness perceptions appear to be important contributors to metabolic control in females, but not males, with type 1 diabetes.

## 1. Introduction

Type 1 diabetes is a national and international health problem and priority, and Norway is amongst the highest incidence of type 1 diabetes in the world. The incidence in Norway has increased by 30% in the last 15 years, and the rate among Norwegian children was 32.5 per 100.000 person-years in 2012 [1]. Only one-third of young patients with type 1 diabetes in the Norwegian Childhood Diabetes Registry (NCDR) manage the target of Hemoglobin A1c (HbA1c, a measure of metabolic control) < 7.5% [2], which is the international target to minimize the risk of developing serious diabetes complications such as cerebrovascular and cardiovascular disease, retinopathy, neuropathy, and nephropathy [3]. Levels of HbA1c are found to predict early and late diabetes complications [4]. Also, childhood-onset type 1 diabetes is associated with increased mortality compared to healthy controls [5].

Type 1 diabetes appears to be a risk factor for the development of eating disorder behaviors. Disturbed eating patterns appear common and persistent in young women with type 1 diabetes, with prevalence rates more than twice those of nondiabetes populations [6, 7]. Although most studies focus on females, some researches suggest that males with type 1 diabetes may also have an elevated risk of developing disturbed eating behaviors [8]. The presence of eating pathology can severely impair metabolic control and advance the onset of long-term complications [9]. A core symptom of disturbed eating in type 1 diabetes is intentional insulin restriction to lose weight, an efficient weight loss strategy uniquely available to patients with type 1 diabetes and reported in up to about 35% of females [10, 11]. Insulin restriction is significantly associated with poorer metabolic control [12], and prior research has found that self-reported insulin restriction leads to a threefold increased risk of mortality at an 11-year follow-up [10].

In addition to disturbed eating behavior, other factors reported to be associated with metabolic control are psychosocial correlates such as illness perceptions [13], coping strategies [14], and insulin beliefs [15]. Illness perceptions refer to individual perceptions or beliefs about their illness and are found to be central to patient behavior in a variety of illnesses, including cancer [16], coronary heart disease [17], and chronic fatigue syndrome [18], as well as type 1 diabetes [19, 20]. Specifically, perceptions of control and consequences have been found to be significantly associated with metabolic control among adolescents and young adults [21]. Coping strategies refer to behaviors adopted to handle negative or stressful events and have been reported to be associated with metabolic control among adolescents and adults [14]. Finally, insulin beliefs refer to patients' beliefs about insulin and have been found to play an important role in adherence to treatment [13, 15] and diabetes control [22].

Although eating disorder psychopathology, coping strategies, illness perceptions, and insulin beliefs have shown to be independently associated with adherence to treatment and metabolic control in patients with type 1 diabetes, little is known about how these variables interact, and how they influence HbA1c, separately and combined. In addition, it is uncertain how these factors operate in males versus females. Increased knowledge may shed light on identifying targets for both assessment and treatment.

### 1.1. Aims of Study

This study aimed to investigate associations between HbA1c and eating disorder psychopathology, coping strategies, illness perceptions, and insulin beliefs in young males and females with type 1 diabetes and to assess the extent to which these variables explain the variance in HbA1c.

## 2. Materials and Methods

### 2.1. Participants and Procedure

The Norwegian Childhood Diabetes Registry (NCDR) is a nationwide, population-based registry, which includes all newly diagnosed children with diabetes since 1989. All pediatric departments in Norway perform and report the results of annual standardized examinations to NCDR. This cross-sectional study is part of a larger study of the NCDR. Of 850 eligible participants, 105 individuals (12%) with type 1 diabetes aged 12–20 years agreed to participate in a more extensive in-depth assessment, including a face-to-face interview.

Participants were compared to the background type 1 diabetes population in the NCDR, which has a completeness of 95% [1]. These groups did not differ on age, zBMI (age- and gender-adjusted body mass index), type 1 diabetes duration, number of consultations with the diabetes team, or number of consultations with dieticians. Participants were slightly older at onset of type 1 diabetes than nonparticipants (9.6 versus 8.8 years, *p* < .05), had somewhat lower HbA1c (8.6% (70 mmol/mol) versus 8.9% (74 mmol/mol), *p* < .05), and had fewer episodes of ketoacidosis (.02 versus .05, *p* < .05).

The participants were recruited from the NCDR between 2011 and 2012 and lived in rural and urban settings across all geographical regions of Norway. There were 44 (41.9%) males and 61 (58.1%) females. Male and female participants did not differ significantly on age (yrs), HbA1c, zBMI, age of onset, or duration of diabetes illness (yrs). The assessment was conducted at Oslo University Hospital or another location at the participants' choice (usually their home or school).

### 2.2. Ethical Aspects

The regional ethics committee approved the study. Written informed consent was obtained from all participants and their parents if the participant was below the age of 16 years.

### 2.3. Measures

The Child Eating Disorder Examination (ChEDE) [23] is a semistructured diagnostic interview that is considered the gold standard for assessing eating disorder psychopathology among children and adolescents. The ChEDE has been translated and validated in Norwegian and has demonstrated good psychometric properties [24]. The ChEDE consists of four subscales (restraint, eating concern, weight concern, and shape concern), in addition to a global score. The answers range from 0 to 6, and higher scores indicate higher degree of eating psychopathology. In line with Olmsted et al. [25], a diabetes-adapted version of the ChEDE was adopted to ensure that pathological scoring was due to weight and shape concerns and not only for controlling the diabetes. As such, any endorsement of items related to eating behavior or food (e.g., food rules and dietary restraint) was further queried to determine whether such behavior was attributable to medically indicated diabetes care or motivated by concerns about their weight and shape. Only attitudes or behaviors motivated by shape/weight concerns were rated. Additionally, after the section on bulimic episodes and overeating was completed, the patient was asked to estimate the percentage of episodes that were associated with a hypoglycemic/low blood sugar reaction. Finally, a separate item for insulin restriction was added, asking whether the patients ever reduced or omitted their insulin dose. If the patients responded affirmatively, they were asked why insulin was restricted. Interviews were conducted by two Masters-level psychologists (Line Wisting and Lasse Bang) who participated in training seminars for the administration of the ChEDE interview by its developer (Rachel Bryant-Waugh). Interrater reliability was assessed for subscales and global score totals and found to be good (composite intraclass correlations coefficient of .97).

Adolescent Coping Orientation for Problem Experiences (A-COPE) [26] is a measure of coping strategies and is translated and validated for use among Norwegian adolescents [27]. In addition to a total score indicating the degree of positive coping, the A-COPE consists of 34 items divided into five subscales: being social, seeking diversions, ventilating negative feelings, developing self-reliance, and solving family problems. Answers range from 1 to 5 (never, seldom, sometimes, often, and usually). Higher scores indicate a higher degree of positive coping on all items, after items 8, 10, 11, 15, 17, 18, 24, and 29 are reversed prior to analyses.

The Brief Illness Perception Questionnaire (BIPQ) [28] is a brief version of the Illness Perceptions Questionnaire (IPQ) [29] and Illness Perceptions Questionnaire-Revised (IPQ-R) [30] and is a valid and reliable measure of illness perceptions. It has been used in the context of a variety of illnesses, including type 1 diabetes. It consists of nine items, and each item assesses one dimension of illness perceptions: consequences, timeline, personal control, treatment control, identity, coherence, emotional representation, concern, and causation. Personal control, treatment control, and coherence are reversed in the analyses, indicating that a higher score on each of the items as well as the total score indicates more threatening/negative views of their diabetes.

The Beliefs about Medicines Questionnaire (BMQ) [31] is a measure of beliefs about medicines in general and one specific medicine (insulin in this study). It consists of four subscales: specific (insulin) necessity, specific concern, general necessity, and general overuse. Answers are ranged on a five-point Likert scale, ranging from 1 = strongly disagree to 5 = strongly agree. A Norwegian version has been translated and validated, demonstrating satisfactory psychometric properties [32]. The specific necessity and specific concern subscales are used in this study to measure participants' concerns regarding insulin and to what extent they perceive insulin to be necessary.

Clinical data were obtained from NCDR. HbA1c was determined for all participants by high-performance liquid chromatography (HPLC) (Tosoh G7; Tosoh Europe N.V., Belgium). All samples were analyzed in the same central laboratory and standardized according to the Diabetes Control and Complications Trial standards. The reference range was 4.0–6.0%; the analytical coefficient of variation was < 1%.

BMI was calculated based on weight and height (kg/m^2^) and standardized to a *z*-score according to age and gender using the Centers for Disease Control and Prevention Growth Charts 2000, since the participants were primarily below 18 years (zBMI) [33].

### 2.4. Statistical Analyses

Associations between metabolic control, eating disorder psychopathology, illness perceptions, coping strategies, and insulin beliefs were assessed by means of Pearson's correlations (*p* < .05). Subsequent to the correlation analyses, standard multiple regression (enter) analyses were conducted in line with the backward elimination strategy described below, to investigate possible risk factors for HbA1c. The analyses were split by gender. Among females, and based on an alpha level of *p* < .20, the BIPQ subscales consequences, personal control, treatment control, identity, coherence, emotional representation, and concern were entered into the equation, in addition to the ACOPE subscale ventilating negative feelings and the ChEDE subscales restraint, eating concern, and weight concern. To avoid multicollinearity in the regression equation, shape concern was excluded due to a correlation with weight concern above .70. Intraclass correlation analysis was used to assess interrater reliability. Statistical analyses were conducted using PASW version 18 (SPSS IBM, NY, USA).

## 3. Results

### 3.1. Participant Characteristics

Mean age of the 105 participants was 15.7 years (SD: 1.8). For males (*n* = 44) and females (*n* = 61), mean age was 15.9 years (SD: 1.8) and 15.6 years (SD: 1.8), respectively. Mean age at onset of type 1 diabetes was 9.6 years (SD: 3.5) for the whole sample, 9.8 years (SD: 3.6) for males, and 9.5 years (SD: 3.5) for females. Mean type 1 diabetes duration for all participants was 5.7 years (SD: 3.7), for males 5.7 (SD: 3.6), and for females 5.7 (SD: 3.7). Regarding age- and gender-adjusted body mass index, mean zBMI was .4 (SD: .8) for the whole sample, .3 (SD: .8) for males, and .4 (SD: .9) for females. Mean HbA1c for the whole sample, males, and females was 8.6% (SD: 1.3), 8.4% (SD: 1.3), and 8.7% (SD: 1.3), respectively. There were no significant differences between males and females on any of the variables.

### 3.2. Correlations

Correlations between HbA1c and illness perceptions, eating disorder psychopathology, coping strategies, and insulin beliefs are presented in Table 1. The correlation analyses were split by gender to investigate gender differences. Among females, HbA1c correlated significantly at the *p* < .05 level with eating restriction (.29, *p* < .05), the illness perception dimensions consequences, personal control, coherence and concern (ranging from .33 to .48), and the coping strategy ventilating negative feelings (−.26, *p* < .05). HbA1c was not significantly associated with any of the variables among males.

### 3.3. Explained Variance in Metabolic Control

Among females, the BIPQ subscales consequences, personal control, treatment control, identity, coherence, emotional representation, and concern were entered into the regression equation, in addition to the ACOPE subscale ventilating negative feelings and the ChEDE subscales restraint, eating concern, and weight concern. This model explained 30% of the variance in HbA1c. After removing nonsignificant variables one by one, only the BIPQ* personal control* dimension remained significant (beta .48, *p* < .001), explaining 23% of the variance in HbA1c among females.

Among males, BIPQ coherence, ACOPE solving problems in the family, ChEDE restriction, ChEDE weight concern, and zBMI were entered into the regression model. This model explained 16.7% of the variance in HbA1c. However, no significant variables remained in the regression equation.

## 4. Discussion

This study investigated associations between HbA1c, eating disorder psychopathology, and psychosocial factors in adolescent females and males with type 1 diabetes. Potential risk factors for poor metabolic control were examined.

Significant gender differences were found in this study. Whereas no variables contributed significantly to the explained variance in HbA1c among males, the overall model including illness perceptions, coping strategies, and eating disorder psychopathology explained 30% of the variance in HbA1c among females. Following the backward elimination strategy, the illness perception* personal control* (“how much control do you feel you have over your diabetes?”) was significantly associated with HbA1c, explaining 23% of the variance. The importance of illness perceptions to metabolic control is also reported in previous research. For example, in Griva et al.'s [34] study of adolescents and young adults (15–25 years) with type 1 diabetes, 30% of the variance in HbA1c was explained by control, consequences, and identity. However, they used the IPQ, which has later been revised (IPQ-R). The more recent IPQ-R and the BIPQ have divided control into personal control and treatment control [28]. The analyses were not separated by gender rendering a direct comparison to our findings difficult. Another study investigated 49 patients with type 1 diabetes above 16 years (mean age 43 years) and found that personal control and identity explained 15% of the variance in HbA1c when using the BIPQ; yet again, males and females were analyzed together. Despite methodological differences in measurement and demographics, however, the results of these studies appear generally consistent with our study. In contrast, McGrady et al. [20] examined illness perceptions as possible predictors of HbA1c in patients with type 1 diabetes (15–25 years) at two time points (baseline and after three months). Contrary to their hypotheses, illness perceptions did not account for significant variance in HbA1c at time 1 or time 2. A discrepancy in findings may be related to assessment differences, as they adopted the Diabetes Illness Representations Questionnaire (DIRQ) rather than a version of the IPQ.

The association between illness perceptions and HbA1c is also apparent when examining the correlation matrix. The BIPQ subscales* consequences*,* personal control*,* coherence,* and* concern* were associated with HbA1c in females (but not in males). More negative perceptions of their diabetes generally indicated higher levels of HbA1c. This is in line with a recent literature review investigating the relationship between illness perceptions and metabolic control across nine studies of adults with type 1 diabetes and type 2 diabetes [19]. Significant associations between HbA1c and the subscales identity, consequences, timeline, concern, and emotional representations were reported when analyzing the nine studies together. However, the associations are considerably weaker than those found among the adolescents in our study. In addition to different age groups and the fact that the review article had a mixed sample of patients with both type 1 diabetes and type 2 diabetes, the divergent results may relate to gender differences. Whereas the current study split the analyses by gender, the other studies investigated males and females together. All the abovementioned studies, including the current study, used some version of the Illness Perception Questionnaire (the Illness Perception Questionnaire, the Illness Perception Questionnaire-Revised, or the Brief Illness Perceptions Questionnaire), facilitating comparison of results. The association between illness perceptions and HbA1c suggests the importance of attention to patients' views and perceptions of their diabetes in clinical settings, especially among females.

HbA1c was associated with eating disorder psychopathology among females. More specifically, HbA1c was associated with the ChEDE subscale eating restriction, but not with the subscales eating concern, weight concern, or shape concern. This makes sense given that eating restriction is the only behavioral subscale of the ChEDE, whereas the remaining three subscales are cognitive. It is only disturbed eating behaviors, not cognitions, which can directly affect metabolic control. However, one might argue that cognitions often lead to specific behaviors, though only indirectly. To our knowledge, no studies have previously investigated the correlation between different ChEDE subscales and HbA1c. However, a couple of studies have reported significant associations between eating pathology total scores and HbA1c with correlation coefficients of .25 [35] and .30 [36]. Both these studies using the self-completion measure the Diabetes Eating Problem Survey-Revised (DEPS-R), which is specifically designed to screen for eating pathology in diabetes. Although few studies have reported correlation coefficients between measures of eating pathology and HbA1c, previous studies have established poorer metabolic control in patients with concurrent type 1 diabetes and disturbed eating than in patients with type 1 diabetes only [9, 12], suggesting an association between these two variables. No significant correlation between HbA1c and eating disorder psychopathology was found among males in our study. This might be expected, since disturbed eating patterns are mostly found among females with type 1 diabetes [6, 7]. Eating disorder psychopathology was hypothesized to affect metabolic control, and in the regression analysis, eating restriction alone explains 8% of the variance in HbA1c in females (not in males). However, this significant effect disappeared when personal control was entered to the regression equation.

Coping strategies have previously been found to be associated with HbA1c [14, 37, 38]. In the current study, higher degree of ventilating negative feelings was associated with higher HbA1c among females. This is in line with previous research, suggesting that emotion-focused coping strategies are associated with poorer metabolic control among adolescents with type 1 diabetes [14]. However, active/problem-focused coping strategies previously shown to be associated with HbA1c in adolescents with type 1 diabetes [14, 37, 38] were not confirmed in this study. Assessment differences with a variety of measures and combination of measures used may account for this discrepancy of findings. In addition, none of these previous studies have separated the analyses by gender, which may complicate comparison of results across the studies.

Insulin beliefs and HbA1c were not found to be significantly associated in this study. Beliefs and perceptions about insulin have been associated with adherence to treatment [13, 15] and diabetes control [22]. However differences in terminology and assessment complicate the interpretation of results. Nevertheless, the importance of attitudes and beliefs about insulin has been emphasized clinically, suggesting that this should be investigated further.

Only one-third of patients in the NCDR manage the international target of HbA1c below 7.5%. This is consistent with international data [39]. Identifying contributing factors to HbA1c levels, and focusing on these clinically, is therefore important. The relevance of illness perceptions was demonstrated among females in this study, suggesting that patient's perceptions of their diabetes and its consequences could be worth addressing in diabetes clinics. In fact, interventions targeting illness perceptions in adults with only type 2 diabetes have successfully improved HbA1c [40], underlining the potential to improve health and minimize the risk of serious diabetes complications. Whether such interventions would yield similar results in adolescents with type 1 diabetes is unknown.

As described above, this study demonstrated several significant associations between psychosocial variables and HbA1c. Common to some of these is that they are relatively small (correlation ranging from .21 to .48). This could question the clinical relevance of the results. However, eating pathology and psychosocial variables explained 30% of the variance in HbA1c in our study, suggesting that such factors are clinically relevant in terms of reducing the risk of diabetes complications.

This study is the last of several studies that recruited patients from the Norwegian Childhood Diabetes Registry (NCDR) at the same time. After participating in previous large-scale studies, 850 patients were invited to participate in this further in-depth assessment of eating disorder psychopathology and psychosocial aspects. A subset of 105 patients agreed to this by actively returning a signed consent form via postal mail. Although only 12% of eligible participants represent a limitation of this study, it is strengthened by the collaboration with the NCDR that ensures clinical data on almost the entire population of young patients with type 1 diabetes in Norway. This enables comparison of participants to nonparticipants on various clinical and demographical data. In general, few significant differences were found between our participants and the nonparticipants, except for somewhat lower HbA1c levels and fewer episodes of diabetes ketoacidosis (see Materials and Methods). This might indicate that our subgroup is slightly healthier than the rest of the population, thereby implying a question of generalizability. Although the differences were relatively small, this might represent a limitation of the current study. The face-to-face assessment conducted with the semistructured diagnostic interview Child Eating Disorder Examination is a strength of this study. This interview is resource-demanding in terms of costs and time given the length of one to two hours.

A majority of existing studies are cross-sectional. These studies, including ours, are limited by their inability to establish causal relations. There is a need for long-term follow-up studies to longitudinally identify risk factors for poor metabolic control and track the development of these among adolescents with type 1 diabetes.

In summary, this study has documented gender differences in the associations between HbA1c, eating disorder psychopathology, illness perceptions, coping strategies, and insulin beliefs. Most important was the association between illness perceptions and HbA1c among females. These issues should be the subject of focus in diabetes clinics as they have shown to affect metabolic control, which is a crucial determinant of serious morbidity and mortality. Further research should investigate potential gender differences in both adolescents and adults. If our findings are replicated in other studies, it is crucial to establish interventions to deal with problematic illness perceptions in type 1 diabetes to improve metabolic control.

## Figures and Tables

**Table 1:** Correlations between HbA1c and illness perceptions (BIPQ), eating disorder psychopathology (ChEDE), coping strategies (ACOPE), and insulin beliefs (BMQ).

	All	Females	Males
BIPQ consequences	.277^*∗∗*^	.355^*∗∗*^	.121
BIPQ timeline	.047	.055	−.006
BIPQ personal control	.365^*∗∗*^	.484^*∗∗*^	.106
BIPQ treatment control	.084	.158	−.091
BIPQ identity	.131	.220	−.133
BIPQ coherence	.155	.328^*∗*^	−.263
BIPQ emotional representations	.177	.219	.048
BIPQ concern	.198^*∗*^	.340^*∗∗*^	−.083
ChEDE eating restriction	.265^*∗∗*^	.287^*∗*^	.188
ChEDE shape concern	.135	.152	−.016
ChEDE weight concern	.191	.172	.205
ChEDE eating concern	.155	.180	−.044
ACOPE being social	.089	.061	.062
ACOPE seeking diversion	−.033	−.009	−.056
ACOPE ventilating negative feelings	−.163	−.260^*∗*^	.034
ACOPE developing self-reliance	−.121	−.135	−.128
ACOPE solving family problems	.089	−.031	.200
BMQ insulin necessity	.035	.080	−.129
BMQ insulin concern	.060	.060	−.016

*Note*. Significance level *∗* = <.05; *∗∗* = <.01.
